# Global analysis of lysine acetylation in soybean leaves

**DOI:** 10.1038/s41598-021-97338-9

**Published:** 2021-09-09

**Authors:** Geng Li, Bin Zheng, Wei Zhao, Tinghu Ren, Xinghui Zhang, Tangyuan Ning, Peng Liu

**Affiliations:** College of Agronomy, Shandong Agricultural University, Tai’an, 271018 Shandong People’s Republic of China

**Keywords:** Biochemistry, Molecular biology, Plant sciences

## Abstract

Protein lysine acetylation (Kac) is an important post-translational modification in both animal and plant cells. Global Kac identification has been performed at the proteomic level in various species. However, the study of Kac in oil and resource plant species is relatively limited. Soybean is a globally important oil crop and resouce plant. In the present study, lysine acetylome analysis was performed in soybean leaves with proteomics techniques. Various bioinformatics analyses were performed to illustrate the structure and function of these Kac sites and proteins. Totally, 3148 acetylation sites in 1538 proteins were detected. Motif analysis of these Kac modified peptides extracted 17 conserved motifs. These Kac modified protein showed a wide subcellular location and functional distribution. Chloroplast is the primary subcellular location and cellular component where Kac proteins were localized. Function and pathways analyses indicated a plenty of biological processes and metabolism pathways potentially be influenced by Kac modification. Ribosome activity and protein biosynthesis, carbohydrate and energy metabolism, photosynthesis and fatty acid metabolism may be regulated by Kac modification in soybean leaves. Our study suggests Kac plays an important role in soybean physiology and biology, which is an available resource and reference of Kac function and structure characterization in oil crop and resource plant, as well as in plant kingdom.

## Introduction

Post-translational modifications (PTMs) are known to regulate many cellular processes and metabolism pathways in dynamic and reversible patterns in biology^1^. With the development of analytical sciences, biochemistry and bioinformatics, increasing novel PTMs have been discovered^2,3^. To date, more than 400 PTMs playing biological processes regulating roles have been reported, such as phosphorylation, ubiquitination, glycosylation and various acylation modifications^4–6^.

In addition, some previous reported PTMs were demonstrated with broaden target substrates and enlarged regulation scope in cellular events and biological processes, and became the research hotspot again, of which lysine acetylation (Kac) was an example. Kac were firstly detected in histones showing potentially RNA synthesis regulation role^7^. However, the latest studies with proteomics techniques and bioinformatics tools in recent years indicated acetylation modification was not limited to histone; a wide range of non-histone proteins were also the substrates for Kac modification^8–10^. Function and pathway analyses have shown Kac involved in various biological processes in both eukaryotes and prokaryotes, such as cellular metabolism (glycolysis, tricarboxylic acid (TCA) cycle, fatty acid metabolism), signal transduction, cell cycle and microtubule stability^8,9,11–13^. Moreover, it has been reported that some disease progressions, especially various cancers, were related to Kac modification^14–17^. Accordingly, some inhibitors of histone deacetylases (HDACs) have been developed for cancer treatment, such as suberoylanilide hydroxamic acid (SAHA) and valproic acid (VPA)^15,18,19^.

In plant kingdom, acetylome in various species and tissues have been studied, especially in model plant species and food crops. In *Arabidopsis*, two relatively early studies found around 100 Kac sites in leaves^20,21^. Subcellular mitochondrion acetylome analysis in *Arabidopsis* found 204 mitochondrion located proteins carrying 243 Kac sites^22^. Then 2057 novel Kac sites corresponding to 959 proteins were discovered in a recent study^23^. Another quantitative acetylome analysis in *Arabidopsis* organs and seedlings reported 909 acetylated proteins, of which 536 acetylated proteins showed changed abundance upon different experiment treatment conditions^24^. In rice, suspension cell, seed, leaves, anthers and whole seedling (root, shoot and leaf) were used as material to uncover the regulation role of Kac in plant physiology and biology; and a wide range of Kac sites and proteins numbers were reported in these studies due to diverse factors^25–31^. The Kac proteins influenced diverse biological and physiological processes of rice, including but not limit to seed germination, seed development and maturation, photosynthesis, carbohydrate metabolism, energy production, protein synthesis, anther development and meiosis^25–31^. Wheat is also a worldwide important food crop, which has been another popular object for acetylome study in various organs and tissues upon diverse experiment conditions^32–34^. In maize, revisable protein acetylation participated in immune response to virulent *C. carbonum*^35^.

Compared with the extensively and thoroughly Kac analysis at proteomics level in model plants and food crops, the acetylome studies in economic plant are relatively less. In strawberry leaves, 1392 Kac sites in 684 proteins were identified^36^. Comparative acetylome study in grape mesocarp and exocarp infected by *Lobesia botrana* (Lepidoptera: Tortricidae) detected 138 Kac sits and demonstrated the role of Kac on plant immune and defense^37^. Other reported species and plant organs/tissues include tea leaves, soybean developing seed, somatic embryos of *P. asperata* and cotton buds, etc^38–41^.

Soybean (*Glycine max*) is one of important economic crops in the world, which serves as a major source of plant proteins and oils^42^. Previous study in developing soybean seed has illustrated Kac involved in RNA synthesis and processing, signaling and protein folding, etc^39^. Leaf is one of indispensable nutrient organ of higher plant, where many central physiological processes occurred, especially photosynthesis. However, the Kac in soybean leaves haven’t been studied. In the presented study, we performed the global Kac identification in the developing leaf tissues of soybean at proteomics level. Various bioinformatics analyses were done in the presented study to reveal the features and roles of Kac in soybean leaf tissues. Our study is of great importance in deeper deciphering for the potential roles of Kac in higher plant leaf physiology, which could serve as an available resource for PTM study in both plant biology and agriculture science.

## Results and discussion

### Detection of Kac in soybean leaf tissues

**Figure 1:**
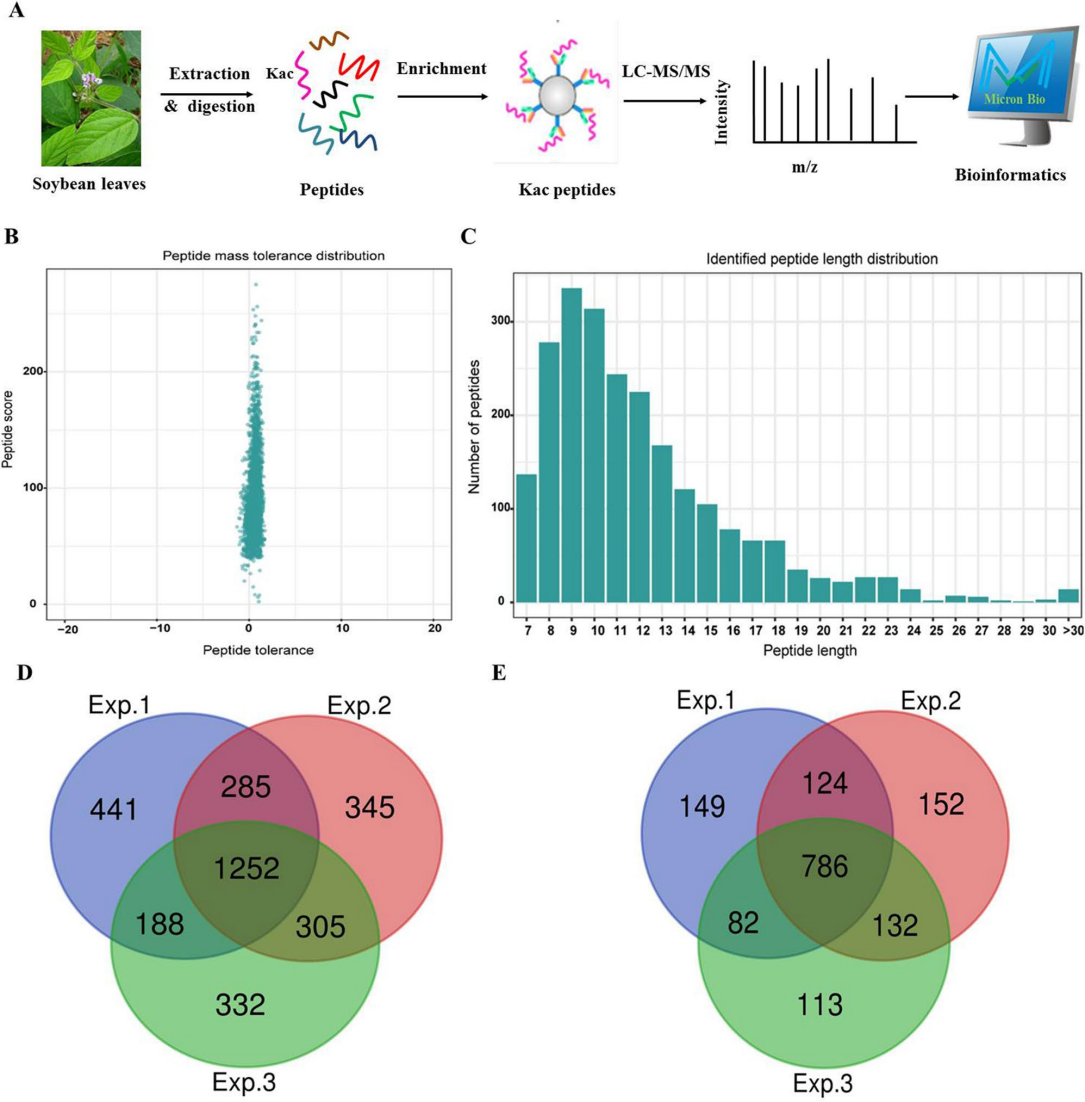
Global analysis of Kac in soybean leaves. (**A**) Experimental procedure for global Kac detection in soybean roots. (**B**) Mass error distribution of all the Kac peptides. (**C**) Peptide length distribution of all the Kac peptides. (**D**) Venn diagram of the identified Kac sites. (**E**) Venn diagram of the identified Kac proteins.

In the presented study, a systematic Kac profiling analysis was carried in the leaf tissues of soybean, with the purpose of revealing the potential roles of Kac in soybean leave growth, development and physiological activities. The general experimental design and workflow was shown in Fig. 1A. In brief, leaves tissues were harvested at blooming, podding and grain filling stages and combined as a replicate. Following that, leaf proteins extraction and trypsin digestion were performed. The resulted peptides were incubated with anti-acetyllysine antibody beads to capture the Kac peptides. Then, the Kac peptides were analyzed by LC–MS/MS. Finally, diverse bioinformatics tools were used to interpret the Kac peptides and proteins. Three biological replicates were performed in the presented study.

A quality control analysis was performed for the acquired MS data. The mass errors of acquired peptides were within 5 ppm, indicating the high accuracy of raw MS data (Fig. 1B). In accordance with the property of tryptic peptides, the majority peptides lengths ranged from 7 to 25 amino acids (Fig. 1C), showing sufficient trypsin digestion. The MS data has met the technically requirements for Kac analysis at proteomics level.

In summary, 3148 Kac sites in 1538 proteins were identified in three biological replicates (Supplementary information Table S1), showing Kac was a rich PTM in soybean leave protein. The Kac sites and proteins identified in each replicate were listed in Table S2, S3 and S4. A Venn analysis was performed to reveal the repeatability within these three replicates (Fig. 1D,E). As the result shown, 1252 (40%) sites in 786 (51%) proteins were repeatedly identified in all the three replicates; and 2030 (64%) sites in 1124 (73%) proteins were repeatedly identified in at least two replicates. The detailed information for these repeatedly identified sites and protein can be seen in Table S1. Only the Kac sites and proteins identified in at least two replicates were used for the following bioinformatics analyses. Previous study in developing soybean seeds detected approximately 400 sites Kac sites in 245 proteins and revealed Kac involved seed development^39^. Our study expanded the dataset of lysine acetylome in soybean species and the enlarged acetylome data facilitated the illustraton of the potential regulation roles of Kac in various other soybean physiological activities. To validate the MS data, a western blotting experiment were performed (Supplementary Information Figure S1). Consistent with the plenty identified Kac proteins in LC–MS/MS analysis, diverse bands with various molecular weights were observed, showing multiple proteins were modified by Kac modification in soybean leaves.

### Motif, amino acid heat map and second structure analyses

**Figure 2:**
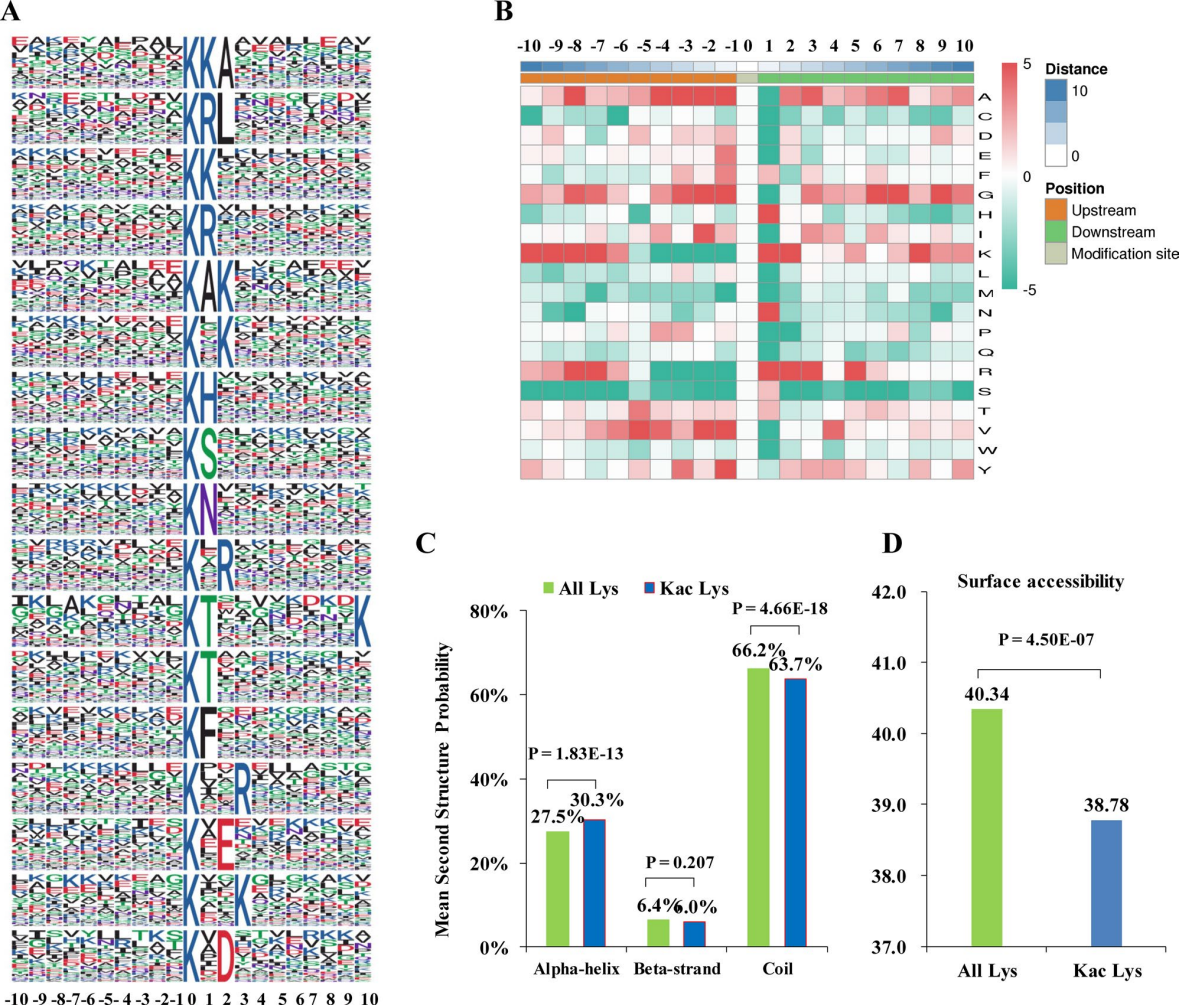
Motif analyses of the detected Kac sites. (**A**) Acetylation sequence motifs for amino acids around Kac sites (− 10 to + 10). The letter height represents the frequency of that amino acid residue at that position. The K in the middle position corresponds to the acetylated lysine. It was performed with motif-x (Version 1.2 10.05.06; https://motif-x.med.harvard.edu/motif-x.html). (**B**) Heat map analysis of the amino acid compositions around the acetylated sites. Red indicates an amino acid that is significantly enriched, while green indicates an amino acid that is significantly reduced. It was performed with the “heatmap.2” function in the “gplots” R-package. (**C**) Secondary structure analysis of Kac peptides. (**D**) Predicted surface accessibility of Kac peptides. Secondary structure and surface accessibility analysis were performed using NetSurfP (version 2.0; http://www.cbs.dtu.dk/services/NetSurfP/).

To characterize the features of the identified Kac peptides, a motif analysis was conducted with Motif-X software (Fig. 2A). A total of 17 conserved motifs were acquired, among which a half motifs contained a conserved lysine (K) or arginine (R) residue at the downstream of Kac sites, such as KacK, KacR, Kac*K and Kac*K (Kac indicated acetylated lysine and * indicates a random other amino acid residue). Besides, 2 acid amino acid containing motifs were enriched, namely Kac*D and Kac*E. Other enriched motifs included KacH, KacN, KacT, KacS and KacF. In agreement with the conserved downstream localized K and R containing motifs, K and R were over-represented at multiples positions surrounding the Kac sites in the heat map analysis for the amino acid composition around the Kac site (Fig. 2B). K was over-presented at − 10 to − 6, + 1, + 2 and + 8 positions while rarely appeared at − 4 to − 1 positions; R was over-represented at − 8, − 7, + 1 to + 3, + 5 positions while scarcely presented − 4 to − 1 positions. The overall frequency of alanine (A) and glycine (G) were much higher than the majority of the rest amino acids at multiple positions, especially at − 4 to − 1, + 6, + 7 and + 9 positions. In addition, valine (V) was significantly enriched at − 7 to − 4, − 2, − 1 and + 4 positions.

Compared with primary structure of Kac proteins in other plant species, such as *Arabidopsis*, rice and tea^23,28,31,43^, we noticed K and R appeared with higher frequency in the consensus sequence motifs in these plant species. Besides, the rest of the conserved motifs identified in our study were also reported in some crop or horticulture species^29,33,36^. Various plant species may share some common conserved motifs surrounding Kac sites.

To evaluate the relationship between protein structure and lysine acetylation in soybean leaves, secondary structure analysis of all acetylated proteins were performed. The result showed 27.5% Kac sites were located in α-helix, 6.4% were located in β-strand and 66.2% Kac sites were located in coil (Fig. 2C). Compared with the distribution of all lysine site, Kac lysine were underrepresented in coil but overrepresented in alpha-helix. In addition, surface accessibility evaluation indicated lysine residues with acetylation modification were relatively less surface-accessible compared with all lysine (Fig. 2D). Kac exhibited a potential protein structure modulation role in soybean leaves.

### Functional classification and subcellular location analysis

**Figure 3:**
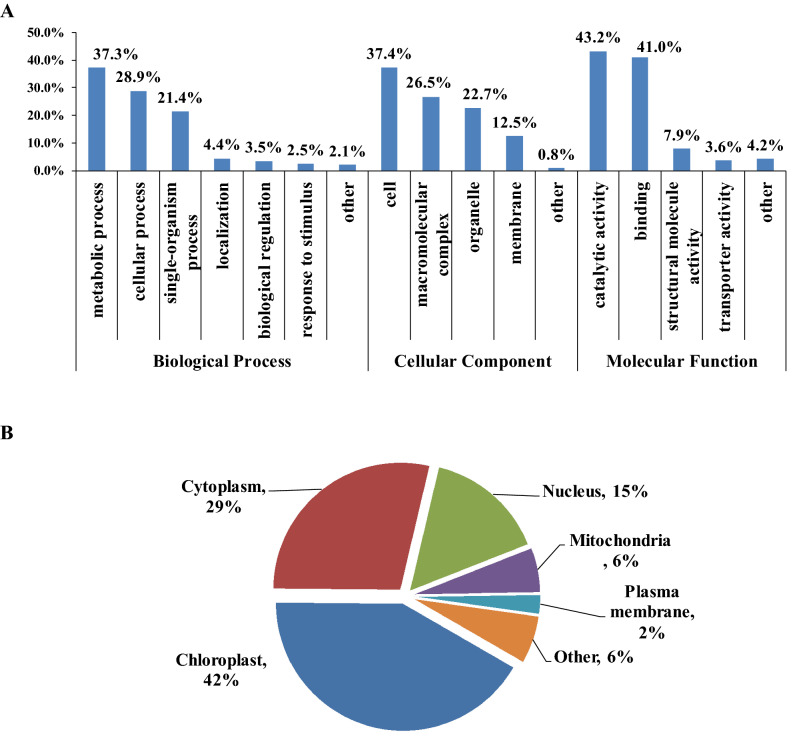
Functional classification and subcellular localization of the Kac proteins in soybean leaves. (**A**) GO annotation based classification. (**B**) Subcellular location predication.

GO annotation based functional classification were implemented to demonstrate the functions of these Kac proteins (Fig. 3A). The classification results for biological processes indicated the top three protein groups of Kac proteins was composed of metabolic process, cellular process and single organism process related proteins, whose percentage was 37.3%, 28.9% and 21.4% in the total Kac proteins, respectively. Other protein groups included localization (4.4%), biological regulation (3.5%), response to stimulus (2.5%) and other (2.1%). Previous studies have reported metabolism and cellular process related proteins were the primary substrates of Kac modification in diverse species including bacteria, fungus, animal and plant; and reversible Kac is an important regulatory pattern in cellular metabolism and cellular homoeostasis^9,44–46^. Our study further evidenced this conception in soybean species.

In term of cellular component, the Kac proteins were mainly classified into the cell (37.4%), macromolecular complex (26.5%), organelle (22.7%), and membrane (12.5%) categories. In the level of molecular function, the result demonstrated that the Kac proteins were primarily associated with binding (43.2%) and catalytic activity (41.0%). Catalyzing is the primary function of enzymes in biochemistry, and binding to the substrates is the physical prerequisite of catalyzing reaction. The high proportion of proteins related to binding and catalytic activity indicates that various metabolic enzymes could be the major protein group that can be modified by Kac. The acetylome study in strawberry leaves and *Picea asperata* somatic embryos also demonstrated the similar perceptive^36,40^. Besides, structural molecular activity (7.9%) proteins were also assigned, implying structure related events within cell such as cell structure maintenance and remodeling, macromolecular complexes structure assembling and disassembling, potentially be adjusted by Kac modification during soybean leave growth and development. The 3.6% transporter activity related proteins hint Kac may involve in molecules transportation in soybean leaves.

GO functional classification result suggested that the Kac proteins in soybean leaves participated in various metabolic and cellular processes; and appeared in diverse cellular components within soybean cell. Binding and catalyzing were the primary molecular events which Kac were involved at molecule category.

The subcellular location predication result (Fig. 3B) indicated the majority of the Kac proteins (42%) were localized to chloroplast, suggesting Kac in this compartment plays a critical role in soybean leaf physiology, which was consistent with previous studies in many other plant leaves^23,28,32,36,38^. Besides, 29% Kac proteins were assigned to the cytoplasm. A number of proteins were also localized to other subcellular locations, such as nucleus (15%), mitochondria (6%) and plasma membrane (2%). The subcellular location prediction of Kac proteins in developing soybean seed indicated cytoplasm and nuclear were the top two locations where Kac proteins were distributed, whose percentage reached approximately 40% and 30%, respectively^39^. The studies in other plant seeds also identified high ratio of cytoplasm and nuclear located Kac proteins^27,34,40^. It seemed that the same tissues/organs (seeds and leaves) in various plant species exhibited similar Kac protein subcellular location distribution. Furthermore, tissue/organ differences may result in relatively higher variation in the subcellular distributions of Kac protein than plant species differences.

### Function and pathway enrichment analysis

**Figure 4:**
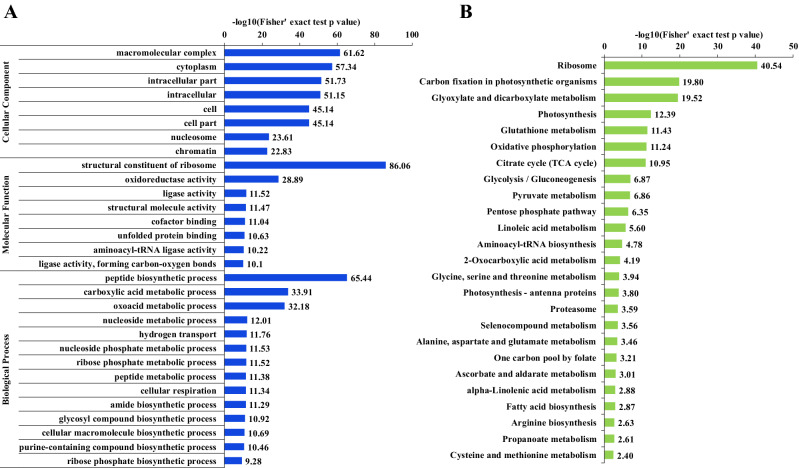
Enrichment analyses of the Kac proteins. (**A**) GO annotation enrichment. (**B**) KEGG pathway enrichment.

To further elucidate the function of these Kac proteins in soybean leaves, GO based function enrichment (Fig. 4A) and KEGG based pathway enrichment (Fig. 4B) analyses were performed. In the category of cellular component, in accordance with the cellular component classification result, a large number of Kac proteins were markedly enriched in macromolecular complex, indicating Kac modification affected the macromolecular complexes within soybean leaf cells. Previous study has illustrated Kac modification preferentially targets large macromolecular complexes participated in multiple cellular processes, such as ribosome complex, chromatin-remodeling complexes, diverse nuclear and cytosolic complexes^10^. The markedly enriched macromolecular complex in soybean leaves in the present study is a well example illustration of this proposition. Cytoplasm and intracellular part were dramatically enriched as well. Other significantly enriched cellular components included cell, cell part, nucleosome and chromatin.

The molecular function enrichment result (Fig. 4A) showed structural constituent of ribosome was the most significantly enriched term, which was consistent with the markedly enriched macromolecular complex term in cellular component analysis and suggested ribosome structure and activity may be influence by Kac modification. Other significantly enriched molecular functions were all enzyme/bioactive molecule activity and binding related terms, matching well with the high parentage binding and catalytic activity related Kac proteins in classification result (Fig. 3A). Corresponding to the molecular function result, the top dramatically enriched peptide biosynthetic process in biological process enrichment further validated the regulation role of Kac in ribosome activity and protein biosynthesis in developing soybean leaves. Lots of material metabolism related processes, especially carbohydrate metabolic processes and energy production processes, were significant enriched as well, such as the metabolic processes of carboxylic acid, oxoacid, nucleoside, nucleoside phosphate and ribose phosphate, hydrogen transport and cellular respiration. Moreover, the enriched molecular biosynthetic processes which involved the biosynthesis of amide, glycosyl compound, cellular macromolecule, purine-containing compound and ribose phosphate implied Kac probably actively participated in the biosynthesis metabolism of various molecules, which laid the foundation for soybean growth and development.

In the KEGG pathway enrichment analysis (Fig. 4B), in accordance with the function enrichment result, ribosome pathway was the top enriched pathway. In addition, aminoacyl-tRNA biosynthesis was also significantly enriched. Translation and protein biosynthesis in soybean leaves may be influenced by Kac modification through Kac mediated ribosome activity regulation and aminoacyl-tRNA biosynthesis. In addition, a number of amino acid metabolism related pathways were dramatically enriched, which involved the metabolism of diverse amino acids such as glutathione, glycine, serine, threonine, alanine, aspartate, glutamate, arginine, cysteine and methionine. Three photosynthesis related pathways were observed, namely carbon fixation in photosynthetic organisms, photosynthesis and photosynthesis-antenna proteins, suggesting photosynthesis process could be affected by Kac modification in soybean leaves. Besides, in parallel with significantly enriched material and energy metabolism related biological processes (Fig. 4A), plenty carbohydrate metabolism and energy production pathways were significantly enriched, such as glyoxylate and dicarboxylate metabolism, citrate cycle (TCA cycle), glycolysis pathways (EMP), gluconeogenesis, pentose phosphate pathway (PPP), gluconeogenesis, pyruvate metabolism and oxidative phosphorylation. What’s noticeable was that some Kac proteins were enriched to three fatty metabolism related pathways: linoleic acid metabolism, alpha-Linolenic acid metabolism and fatty acid biosynthesis. Kac modification possibly played a role in fatty acid biosynthesis and fatty accumulation in this important oil crop.

Taken together, the wide distribution of Kac proteins in enrichment analysis suggested Kac modification played an important regulatory role in diverse physiological processes and metabolism pathways in soybean leaves, especially in ribosome activity and protein biosynthesis, photosynthesis, glycometabolism and fatty acid metabolism.

### Protein–protein interaction (PPI) analysis

**Figure 5:**
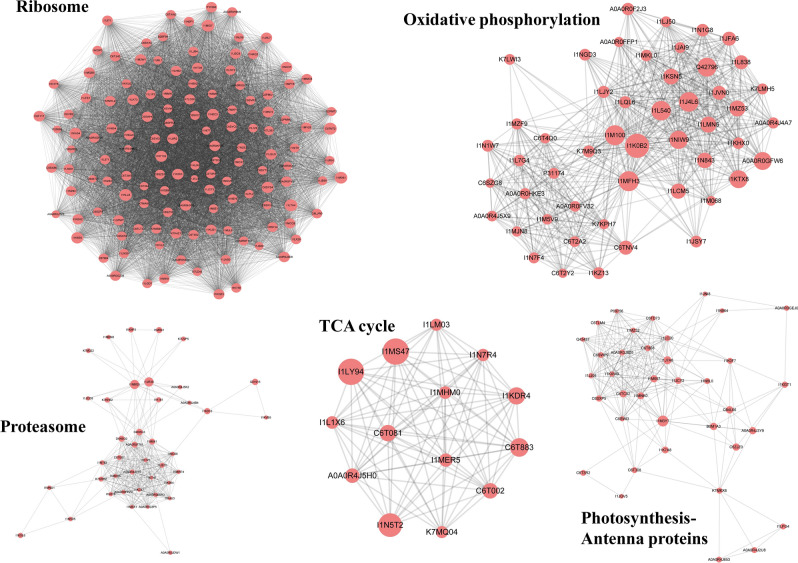
PPI network analysis of the Kac proteins. Five highly-connected clusters were presented.

To investigate the interactions and the cross-linked pathways among these Kac proteins, the PPI network was established for all of the Kac proteins in soybean leaves. Totally, 1503 Kac proteins were mapped to the search tool to acquire PPI network (Supplementary Information Figure S2). By using the MCODE plug-in tool kit, we retrieved 5 highly-connected clusters from the overview network, which included ribosome, oxidative phosphorylation, proteasome, TCA cycle and photosynthesis-antenna proteins (Fig. 5). The extracted clusters ribosome and proteasome was in agreement with the dramatically enriched protein metabolism related GO terms and KEGG pathways (Fig. 4A,B) and further evidenced the potential regulation role of Kac modification in ribosome activity and protein biosynthesis. In addition, the rest three functional modules, TCA cycle, oxidative phosphorylation and photosynthesis-antenna proteins were also enriched in the preceding KEGG pathways enrichment analysis, which implied the possible regulatory effects of Kac modification on these physiological processes in growing soybean leaves.

### Kac regulated ribosome activity and protein biosynthesis

The most significantly enriched ribosome pathway (Fig. 4A) and ribosome cluster (Fig. 5) suggested ribosome activity probable be influenced by Kac modification. A total of 336 sites on 50 ribosome related subunits or components were detected (Table S5). Kac modification has been reported participated in PPI through diverse mechanisms^11^. The Kac sites on these subunits/components may influence the interactions among these subunits/components and further regulate the overall ribosome activity and protein biosynthesis. Previous acetylome studies in the leaves of wheat, rice and strawberry also detected ribosome related Kac protein^28,32,36^; while was less significant compared with soybean leaves. Species variance perhaps resulted in this difference as soybean protein is an important source of plant protein while the primary nutrients in cereal or fruit were starch or carbohydrates^47,48^; thus proteins biosynthesis is more active in soybean, especially at grain filling stage. According to previous reports, some proteins synthesized in soybean leaves, such as vegetative storage proteins (VSPs), serve as temporary storage molecules for nitrogen^49,50^. They can be rapidly synthesized and subsequently broken down; and the resulted amino acids or metabolites are presumably exported to developing seeds via the phloem transport system to support seed protein synthesis^49,50^. In soybean, translocation from vegetative tissue (mainly leaves) contributes no less than 50% of the fixed nitrogen utilized by seeds^49–51^. The Kac modification could potentially influence the biosynthesis of VSPs in soybean leaves, and further mediate soybean seed protein synthesis and accumulation. Apart from ribosome activity regulation, aminoacyl-tRNA biosynthesis is another potential pattern of Kac regulated protein biosynthesis in soybean leaves, which have been reported in many other plant species as well^29,31,38^. A number of amino acid metabolism related pathways (Fig. 4B) suggested Kac participated in amino acid metabolism and acted as a protein biosynthesis control role at substrates level. The study in developing soybean seeds matched high percentage protein metabolism related Kac proteins (25%) involved in protein synthesis, folding, targeting and storage^39^. Proteins metabolism, especially ribosome activity and protein biosynthesis is a primary Kac modification mediated cellular process and pathway in soybean.

### Kac regulated photosynthesis

Previous study in *Cyanobacterium Synechocystis* identified 33 Kac proteins involved in photosynthetic pathway and many of them were aligned to protein complexes within photosynthesis system, such as photosystem II (PSII) and photosystem I (PSI)^52^. In rice, wheat, *Arabidopsis* and tea leaves, Kac modification mediated light reaction related processes have been demonstrated as well^23,28,32,43^. The enriched photosynthesis-antenna protein related pathway (Fig. 4B) and functional model (Fig. 5) in the presented study implied photosynthetic light reaction possibly were influenced by Kac modification in soybean.

Apart from light reaction, a lot of dark reaction related proteins and enzymes were detected Kac modified in multiples sites; almost all the members belonging to Calvin cycle underwent Kac modification (Table S5), which further convinced Kac modification is a conversed carbon fixation regulatory pattern in species having photosynthesis ability^23,28,32,36,43^.

### Kac regulated carbohydrate and energy metabolism

Carbohydrate metabolism and energy production is of vital significance in plant biology as it serves as the material and energy foundation for almost every physiological activity^53^. Our study showed the central carbohydrate metabolism pathways, including EMP, TCA and PPP may be influenced by Kac modification in soybean leaves (Fig. 4B). The majority of enzymes involved in these pathways were Kac modified in multi sites (Table S5), such as malate dehydrogenase (5 sites), citrate synthase (5 sites), 6-phosphogluconate dehydrogenase (5 sites), glyceraldehyde-3-phosphate dehydrogenase (7 sites) and phosphoglycerate kinase (8 sites). Previous studies in bacteria and mammal have well illustrated Kac participated central carbohydrate metabolism, especially TCA cycle and EMP pathways^8,9,54^; and Kac mediated central carbohydrate metabolism is highly evolutional conserved from bacteria to mammal^46,54,55^. It seemed that the demonstration of Kac modification in PPP is lacking in bacterial, yeast and mammal. On the contrary, the reports which pictured Kac affected PPP is relatively abundant in plant species^26,30^. Our study in soybean leaves found a number of enzymes aligned to PPP (Table S5), such as fructose-bisphosphate aldolase, ribulose-phosphate 3-epimerase, 6-phosphogluconate dehydrogenase and transketolase_1 domain-containing protein, which further proved Kac affected PPP in plants.

**Figure 6:**
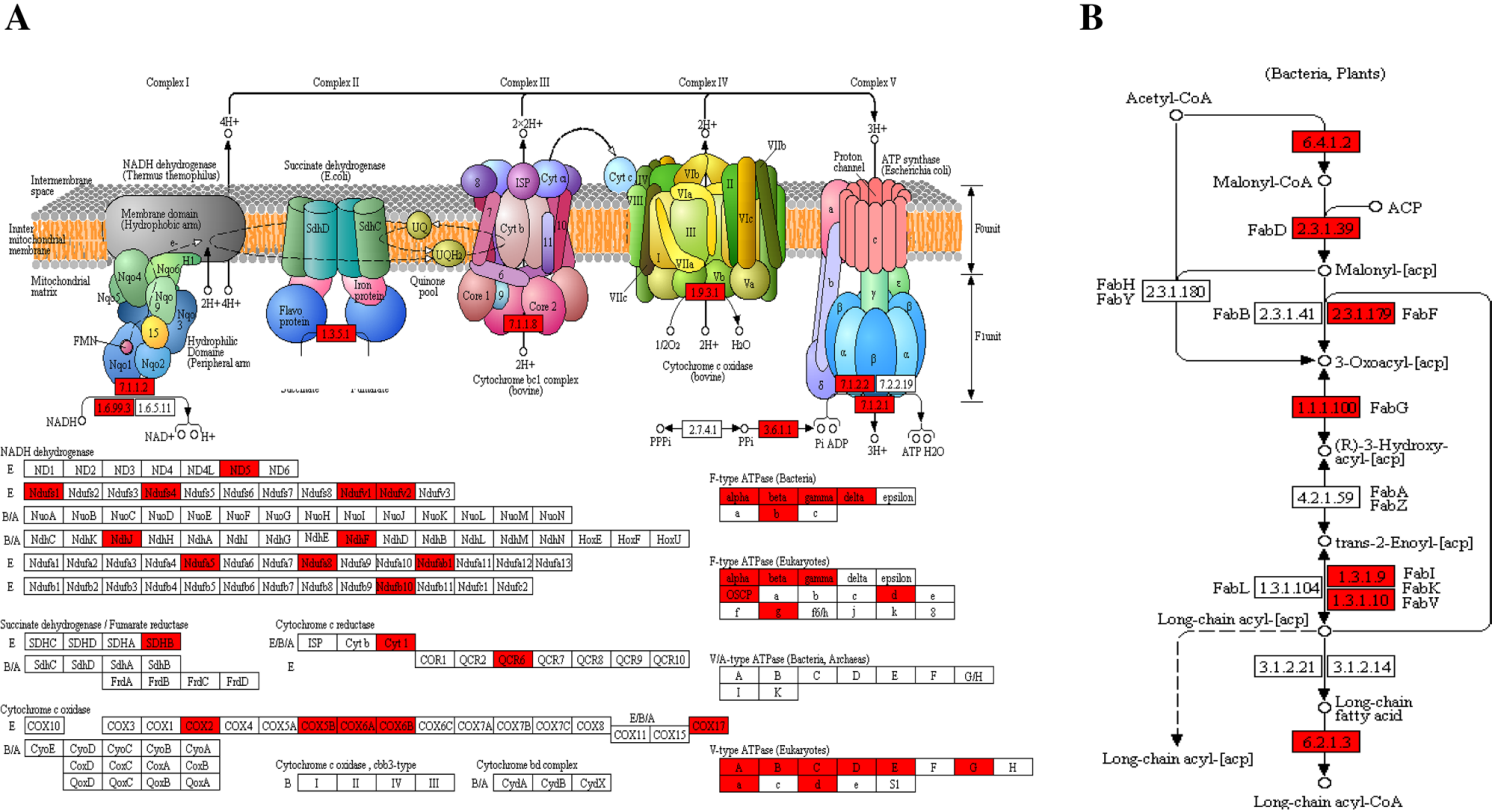
Representative significantly enriched KEGG pathways. (**A**) Oxidative phosphorylation. (**B**) Fatty acid biosynthesis. The Kac proteins are labeled in red. The figures were prepared with referring KEGG pathway map images with permission.

Oxidative phosphorylation resulted ATP accumulation is critical in plant due to its higher energy production efficiency. The dramatically enriched oxidative phosphorylation pathway (Fig. 6A) indicated Kac modification occurred in all the five protein complexes in the oxidative phosphorylation electron transfer chain. The acetylation moiety on the subunits or domains of these protein complexes probably affected the molecular interactions within complex or between complexes, and further influenced the overall structure and functional activities of these complexes. The markedly extracted oxidative phosphorylation functional module (Fig. 5) evidenced this speculation.

### Kac mediated fatty acid metabolism

Soybean is an important oilseed crop grown worldwide^47^. The study on soybean oil yield enhancement and oil quality improvement is a critical issue in agriculture science^47,48^. In the present study, the dramatically enriched fatty acid biosynthesis, linoleic acid metabolism and alpha-Linolenic acid metabolism pathways (Fig. 4B) indicated Kac may involve in fatty acid biosynthesis and fatty accumulation in soybean. As shown in Fig. 6B, almost all the enzymes belonging to fatty acid biosynthesis pathways were Kac modified. Besides, linoleic acid is highly associated with soybean oil flavor and quality, implying Kac modification may influence soybean oil quality through regulating the enzymes activities involved in linoleic acid metabolism. We infer some metabolic intermediates of fatty acid metabolism in soybean leaves may be transported into seed; and participated in seed fatty acid biosynthesis and oil accumulation. Further study is needed to demonstration this hypothesis. Adjusting the Kac level of these fatty acid metabolism related enzymes with biotechnology is of potential significance in oil crop science.

### Conclusion

In the present study, the systematic Kac in soybean, an important oil crop and industrial crop, was performed with leaf tissues. The results indicated Kac was a rich PTM in soybean leaf proteins. These Kac proteins were distributed to diverse subcellular locations, of which chloroplast located proteins accounted the highest ratio. Function and pathway analyses showed Kac participated in multiple cellular processes and metabolic pathways, whose mechanisms at molecular level were mainly concerned with catalyzing and binding. Ribosome activity and protein biosynthesis related proteins are the most preferred substrates of Kac modification in soybean leaves. Besides, carbohydrate metabolism and energy production and photosynthesis may be regulated by Kac modification. Moreover, the enriched fatty acid metabolism related pathways imply fatty biosynthesis and accumulation is potentially be influenced by Kac. Our study expanded the scope of Kac in plant species, especially in resource plants, and could serve as a reference of the further Kac study in oil plants, as well as in other crop plants.

## Materials and methods

### Soybean cultivation

The soybean cultivar 34 was used for the experiment. The soybean cultivation and material collection was performed in an experimental field of Shandong Agricultural University in Tai’an (36° 9′ 40″ N, 117° 9′ 48″ E), Shandong Province, China. The general climate indicators were listed as follows: the average annual temperature was 13 °C; the average annual amount of sunshine was 2600 h; the mean rainfall was around 700 mm. The soybean seeds were sowed in the field and the type soil was Typic-Hapli-Udic Argosols based on Chinese Soil Taxonomy^56^. Normal fertilizers and water supplied and the plants were protected from pests during the whole cultivation stage. The leaf tissues were harvested at the stages of blooming, podding and grain filling, respectively. Only the top three freshly grown leaves in the upper part of the plants at each stage were collected and stored at − 80 °C. Three biological replicates were prepared for each stage. The collection of plant specimens has been approved by College of Agronomy, Shandong Agricultural University, Tai’an City, P. R. China. Experimental research and field study on soybean in this study has complied with the IUCN Policy Statement on Research Involving Species at Risk of Extinction.

### Protein extraction and trypsin digestion

The leaves harvested at different stages were mixed with equal amount and used as a biological replicate. Then the leaves were grinded into powder with liquid nitrogen and homogenized in solution containing 8 M urea, 1% Triton-100, 10 mM dithiothreitol (DTT), and 1% Protease Inhibitor Cocktail. After 10 min sonication on ice, cell debris were removed through centrifugation at 15 000 g for 20 min at 4 °C. Then the supernatant was precipitated with ice-cold acetone for more than 4 h at − 20 °C and then centrifuged at 15 000 g for 20 min at 4 °C. Following three times cold acetone washes and air drying, the protein pellets were re-suspended in 8 M urea. The resulted suspension was reduced with 5 mM DTT for 30 min at 56 °C following alkylating with 11 mM iodoacetamide for 15 min at room temperature in darkness. After dilution with 100 mM Triethylammonium bicarbonate buffer (TEAB) to reduce urea concentration to less than 2 M, a two-step trypsin digestion was carried out according to the method of Zhang et al.^32^. After digestion, peptide was desalted by Strata X C18 SPE column (Phenomenex) and vacuum-dried.

### Affinity enrichment for Kac peptides

For affinity enrichment, the peptide were incubated with pre-washed pan anti-acetyllysine antibody beads (WM Hangzhou, China) in NETN buffer (100 mM NaCl, 1 mM EDTA, 50 mM Tris–HCl, 0.5% NP - 40, pH 8.0) at 4 °C overnight with gentle shaking. After washing four times with NETN buffer and twice with double distilled water, the lysine acetylation peptides bound to the agarose beads were eluted with 0.1% trifluoroacetic acid. Finally, the eluted fractions were combined and vacuum-dried for further use.

### LC–MS/MS analysis

The dried peptides were firstly dissolved in 0.1% formic acid (FA) and separated using a reversed-phase analytical column ((Thermo Acclaim PepMap RSLC C18 column, 2 μm, 75 μm × 50 mm) on an EASY-nLC 1000 UPLC system. Then, the peptides were subjected to NSI source followed by tandem mass spectrometry (MS/MS) in Q Exactive (Thermo Scientific) coupled online to the UPLC system. The voltage for electrospray analysis was set at 2.0 kV. Detection of intact peptides were performed in the Orbitrap at a resolution of 70,000 with Normalized Collision Energy (NCE) setting of 25. For MS scan, the m/z range was set from 350 to 1800. A data-dependent procedure that alternated between one MS scan followed by 15 MS/MS scans was applied for the top 15 precursor ions above a threshold ion count of 1E5 in the MS survey scan with 15.0 s dynamic exclusion. Automatic gain control (AGC) was used to prevent overfilling of the Orbitrap; 5E4 ions were accumulated for generation of MS/MS. LC–MS/MS analysis was performed blindly by Micrometer Biotech Company (Hang zhou, China).

### Database Searching

The aacquired MS raw data was searched using Maxquant search engine (version.1.5.2.8) against the *Glycine max* database from Uniprot (74863 sequences, https://www.uniprot.org/proteomes/UP000008827), which concatenated with reverse decoy database and common proteins contaminants. False discovery rate (FDR) thresholds for Kac peptides and proteins were specified as 1%. Trypsin/P was used as cleavage enzyme with up to 2 missing cleavages and set the minimum number of amino acids as 7. Kac site localization probability was set as > 0.75. Carbamidomethylation on cysteine was specified as fxed modifcation and oxidation on methionine, acetylation on lysine and protein N-terminal were specified as variable modifications. Mass error was set to 6 ppm for precursor ions and 0.02 Da for fragment ions. All the MS data were deposited to ProteomeXchange Consortium via the PRIDE partner repository. The accession number is PXD021246.

### Bioinformatics analysis

Motif analysis for Kac peptides was performed with Motif-X software; all the database protein sequences were used as background, other parameters were set with default^57^. Gene Ontology (GO) based annotation was used for the functional classification and functional enrichment analysis^58^. The Kyoto Encyclopedia of Genes and Genomes (KEGG) database based pathway annotation was used for pathway enrichment analysis^59^. GO and KEGG enrichment analyses were carried out with DAVID tool with the corrected *p* value < 0.05^60^. Subcellular location analysis was conducted with Wolfpsort software^61^. We used Cytoscape software (v3.7) to analyze protein–protein interactions (PPI) of identified proteins^62^. The PPI network was obtained from the STRING (v11.0) database with the interaction confidence score ≥ 0.7 (high confidence) and molecular complex detection (MCODE) plug-in tool was utilized to extract highly enriched interaction clusters^63^.

## Supplementary Information

Supplementary Information.
